# Daily Rhythms of Hunger and Satiety in Healthy Men during One Week of Sleep Restriction and Circadian Misalignment

**DOI:** 10.3390/ijerph13020170

**Published:** 2016-01-29

**Authors:** Charli Sargent, Xuan Zhou, Raymond W. Matthews, David Darwent, Gregory D. Roach

**Affiliations:** Appleton Institute for Behavioural Science, Central Queensland University, P.O. Box 42, Goodwood 5034, Australia; x.zhou@cqu.edu.au (X.Z.); r.w.matthews@cqu.edu.au (R.W.M.); david.darwent@cqu.edu.au (D.D.); greg.roach@cqu.edu.au (G.D.R.)

**Keywords:** sleep restriction, hunger, satiety, core body temperature, visual analogue scales, forced desynchrony

## Abstract

The impact of sleep restriction on the endogenous circadian rhythms of hunger and satiety were examined in 28 healthy young men. Participants were scheduled to 2 × 24-h days of baseline followed by 8 × 28-h days of forced desynchrony during which sleep was either moderately restricted (equivalent to 6 h in bed/24 h; *n* = 14) or severely restricted (equivalent to 4 h in bed/24 h; *n* = 14). Self-reported hunger and satisfaction were assessed every 2.5 h during wake periods using visual analogue scales. Participants were served standardised meals and snacks at regular intervals and were not permitted to eat *ad libitum*. Core body temperature was continuously recorded with rectal thermistors to determine circadian phase. Both hunger and satiety exhibited a marked endogenous circadian rhythm. Hunger was highest, and satiety was lowest, in the biological evening (*i.e.*, ~17:00–21:00 h) whereas hunger was lowest, and satiety was highest in the biological night (*i.e.*, 01:00–05:00 h). The results are consistent with expectations based on previous reports and may explain in some part the decrease in appetite that is commonly reported by individuals who are required to work at night. Interestingly, the endogenous rhythms of hunger and satiety do not appear to be altered by severe—as compared to moderate—sleep restriction.

## 1. Introduction

Across the developed world, the number of individuals undertaking shiftwork continues to increase. In Australia, 16% of the working population is employed in shiftwork [1] and slightly higher figures (~20%) have been reported for Europe [2] and the United States [3]. Shiftwork is associated with several health problems, including obesity, diabetes, and cardiovascular disease [4,5,6]. Numerous factors are thought to contribute to the increased risk of metabolic disorders observed in individuals who undertake shiftwork, including misalignment between the endogenous circadian timing system and behavioural cycles of sleep/wake and fasting/feeding [7,8].

In healthy adults, most physiology and behaviour follow reproducible oscillations across the 24-h day. These rhythmic processes are governed by an internal circadian timing system but are also modulated by exogenous factors such as the light-dark cycle, social demands, and work schedules [9,10,11]. In a recent study, Scheer *et al.* [12] examined whether the internal circadian timing system exerts an influence on sensations of hunger in healthy adults with the use of a 20-h forced desynchrony protocol. The advantage of this protocol is that sleep periods and meals can be tightly controlled and perceptions of hunger can be assessed across the entire circadian cycle. Indeed, a marked endogenous rhythm in hunger was observed, that peaked in the evening (~19:50 h) before gradually declining to its lowest level in the morning (~07:50 h).

A key feature of the forced desynchrony protocol employed by Scheer *et al.* [12] was that it maintained a normal ratio of sleep to wake (*i.e.*, 1:2), such that participants spent one-third of each experimental day in bed and remained awake for the other two-thirds. However, most individuals who undertake shiftwork typically obtain only 5–6 h of sleep [13]. This is an important issue because there are data to suggest that the influence of the circadian timing system on physiological and behavioural function is altered when the ratio of sleep to wake is reduced. For example, Zhou *et al.* [14,15] demonstrated greater impairments in neurobehavioural performance and subjective alertness during the biological night compared with the biological day when the ratio of sleep to wake was reduced in a standard forced desynchrony protocol from 1:2 (equivalent to 8 h sleep per 24 h) to 1:5 (equivalent to 4 h per 24 h). It therefore seems plausible that the influence of the circadian timing system on perceptions of hunger may also be altered when sleep is restricted (*i.e.*, <8 h of sleep per 24 h) [16]. The aim of the present study was to examine the impact of the circadian process on perceptions of hunger and satiety using a forced desynchrony protocol in which the ratio of sleep to wake was either moderately or substantially lower than normal (*i.e.*, 1:3 and 1:5, respectively).

## 2. Materials and Methods

### 2.1. Participants

Twenty-eight male volunteers with a mean (±SD) age of 22.7 (±3.9) years and a mean body mass index of 22.8 (±2.2) kg/m^2^ gave written, informed consent to participate in the study. The participants were in good mental and physical health as determined by in-person interviews and responses to several screening questionnaires. They were free of neurological diseases, psychiatric disorders, endocrine disorders, and sleep disorders. The participants did not consume large doses of caffeine (no more than 350 mg/day) or alcohol (no more than six standard drinks/week); they were non-smokers, medication free and had not undertaken shift work or flight across more than two time zones in the three months prior to the study. Ethics approval for the study was granted by the University of South Australia Human Research Ethics Committee (P301/07) using guidelines established by the National Health and Medical Research Council of Australia and the study was conducted in accordance with the principles of the Declaration of Helsinki.

### 2.2. Prospective Consumption, Hunger, Satisfaction and Fullness

Visual analogue scales were used to measure prospective consumption, hunger, satisfaction, and fullness [17]. The scales were 100 mm long and anchored with opposing phrases related to the construct as follows:

- Prospective consumption: “How much do you think you could eat?” anchored with “nothing at all” and “a lot”;
- Hunger: “How hungry do you feel?” anchored with “I am not hungry at all” and “I have never been more hungry”;
- Satisfaction: “How satisfied do you feel?” anchored with “I am completely empty” and “I cannot eat another bite”;
- Fullness: “How full do you feel?” anchored with “not at all full” and “totally full”.

Visual analogue scales have been shown to be sensitive to experimental manipulations and are a useful adjunct to measures of food, energy and nutrient intake [18]. In addition, they show good reproducibility under controlled conditions when used in within-subjects designs, as is the case in the present study [17].

### 2.3. Core Body Temperature and Physical Activity

Core body temperature (CBT) was continuously sampled at 1-min intervals with a rectal thermistor (Cincinnati Sub-Zero Products, Cincinnati, OH, USA) connected to a data-logger (Minimitter, Bend, OR, USA). Physical activity was continuously measured in 1-min epochs using wrist activity monitors (Philips Respironics, Andover, MA, USA) fastened to the non-dominant wrist.

### 2.4. Circadian Phase Estimates

The generation of phase estimates from the CBT data was a five-step process that involved: (i) cleaning the raw CBT data to account for erroneous or missing values due to downloading of the data, slippage of the thermistor, or malfunction of the equipment; (ii) demasking for physical activity using a purification by intercepts approach [19]; (iii) demasking for sleep-wake differences using a sleep-state correction factor; (iv) fitting of a cosine equation with a fundamental period (24 h = 360°) and a single harmonic to the demasked CBT data using the method of least squares; and (v) assigning a circadian phase estimate (*i.e.*, 0°–360°) to each minute of the beat period using the resultant cosine equation (for more details on this five-step process, see [20]). This process has been used in previous studies to generate phase estimates from CBT data (e.g., [14,15,20,21,22,23,24].

### 2.5. Laboratory Conditions

The study was performed in a time-isolation sleep laboratory in Adelaide, Australia. The laboratory is windowless, sound-attenuated, and temperature-controlled, and was kept free of all time cues during the study. The laboratory was configured such that it could accommodate three participants at a time, each with their own bedroom, living room and bathroom facilities. The laboratory also includes a kitchen and dining room. Ambient light levels during wake periods were <15 lux at the angle of gaze at a height of 183 cm from the floor. During sleep periods, all lights were extinguished (*i.e.*, <0.03 lux). The target room temperature was 22 (±1) °C. The participants were not permitted to leave the laboratory at any time during the study.

### 2.6. Protocol

**Figure 1:**
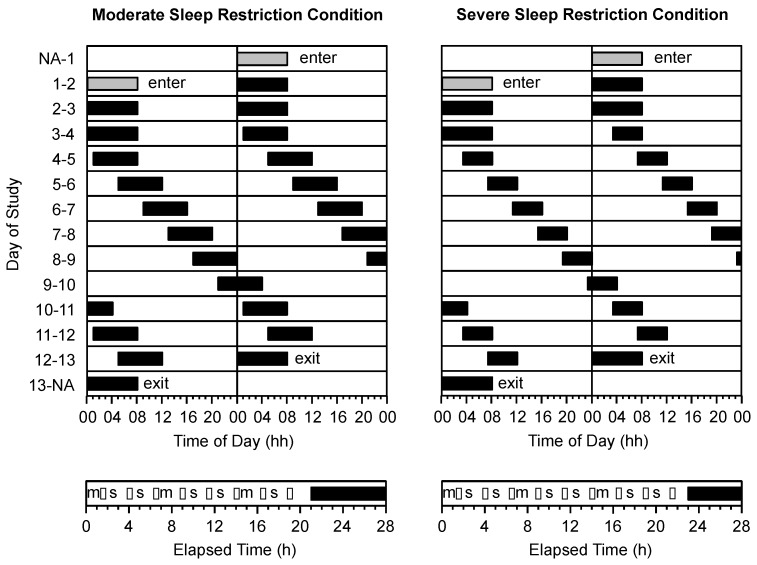
Double raster plot of the study protocol in the moderate sleep restriction condition (**top left panel**) and the severe sleep restriction condition (**top right panel**). The vertical axis represents calendar day of study. The horizontal axis represents the time of day and is 24 h in length. Grey bars represent time in bed on baseline days. Black bars represent time in bed on forced desynchrony days in the moderate sleep restriction condition (equivalent to 6 h time in bed/24 h) and the severe sleep restriction condition (equivalent to 4 h time in bed/24 h). The timing of (m) meals, (s) snacks and (•) test batteries are illustrated as a function of elapsed time into the wake period in the moderate sleep restriction condition (**bottom left panel**) and the severe sleep restriction condition (**bottom right panel**).

The protocol required participants to live in the sleep laboratory for 11 consecutive days. Participants completed either a moderate sleep restriction condition (*n* = 14; age = 23.6 ± 4.0 years) or severe sleep restriction condition (*n* = 14; age = 21.8 ± 3.8 years). In both conditions, the protocol consisted of 2 × 24-h days of baseline followed by 8 × 28-h days of forced desynchrony (Figure 1). On the baseline days, all participants had 16 h of wake followed by 8 h in bed (*i.e.*, 00:00 to 08:00 h). On forced desynchrony days, participants in the moderate sleep restriction group had 21.0 h of wake followed by 7.0 h in bed (equivalent to 6 h time in bed/24 h) and participants in the severe sleep restriction group had 23.3 h of wake followed by 4.7 h in bed (equivalent to 4 h time in bed/24 h). Throughout the protocol, time in bed was scheduled such that wake periods began at the same time for participants in both conditions. During the forced desynchrony phase of the protocol, this meant that bedtimes for participants in the severe sleep restriction group were delayed by 2.3 h relative to the bedtimes for participants in the moderate sleep restriction group. Wake times were matched so that neurobehavioral testing (see below) could occur at the same level of prior wake, and at the same time of day, in both groups.

### 2.7. Procedure

During wake periods, participants completed a 1-h neurobehavioral test battery every 2.5 h, with the first test battery beginning 1.5 h after wake-up time (*i.e.*, at 1.5, 4, 6.5, 9, 11.5, 14, 16.5, 19 h into each wake period; Figure 1—lower panels). Participants in the severe sleep restriction group completed an additional test battery (*i.e.*, at 21.5 h into each wake period), however the data from this test battery were not included in the analyses. The test battery included visual analogue scales for prospective consumption, hunger, satisfaction, and fullness. The test battery was performed in the participants’ bedrooms and the tests were completed in the same order each time. A range of other tasks was included in the test battery (e.g., psychomotor vigilance task, driving simulator, *etc.*), the results of which are reported elsewhere [14,15,20,21,22,23,24].

During sleep episodes, participants remained in bed with lights off until the scheduled wake-up time, unless they needed to use the bathroom. At the end of each sleep episode, the participants were given 15 min to shower and prepare for the “day” ahead. Participants were not permitted to sleep at any time during wake periods. Researchers monitored participants’ compliance with the protocol, either in person or via the closed circuit television system.

The participants were not completely isolated from one another, or from the researchers, during wake periods, but their social interaction was minimised. In particular, the majority of the participants’ free time between test batteries was spent in their own private lounge rooms reading books, watching movies, drawing, or listening to recorded music. The main interactions between participants occurred at meal times when they ate together in a communal dining room, and the main interactions between participants and researchers occurred during administration of the test batteries and serving of meals.

### 2.8. Meals

During the study, participants were not permitted to eat *ad libitum*. Participants were served three standardised meals (*i.e.*, breakfast, lunch, dinner) at the same elapsed time into each wake period (*i.e.*, 0.5 h, 7.5 h, 15 h, respectively; Figure 1—lower panels). The macronutrient content of each meal approximated that of a habitual western diet (*i.e.*, 50% carbohydrate, 30% fat, 20% protein) [25]. Breakfast consisted of a choice of toast, fruit, yoghurt and cereal; lunch consisted of a choice of salad and cold meats for sandwiches, soup or noodles; and dinner consisted of a hot meal of rice or pasta served with meat and/or vegetables. The participants in both conditions consumed all of their main meals. In addition to main meals, five small snack opportunities were provided at the same elapsed time into each wake period (*i.e.*, 2.5 h, 5 h, 10 h, 12.5 h, 17.5 h; Figure 1—lower panels). Participants in the severe sleep restriction group were provided with one additional snack opportunity (*i.e.*, 20 h). Participants could choose one of the following items for their snack: 1 × 31.3 g muesli bar, 2 × 18 g sweet biscuits, 1 × 19 g packet of potato chips, 1 × 25 g packet of savoury crackers, 1 × piece of seasonal fresh fruit, or 1 × 40 g packet of dried fruit and nuts. The data relating to snack consumption and snack choice have been reported previously [24]. Water was made available to participants at all times.

### 2.9. Statistical Analyses

For all measures (*i.e.*, prospective consumption hunger, satisfaction, and fullness), individual data points were assigned a circadian phase and a time since last meal. For analysis of the circadian component, a bin width of 60° (~4 h) was selected. For analysis of the time since last meal component, three levels were identified that corresponded to the elapsed time since the initiation of the last meal (*i.e.*, 1.5 h, 4 h, 6.5 h). Time since last meal was chosen—rather than time since last snack—because meals were the participants’ primary source of calories. To ensure that each participant contributed equally to subsequent analyses, data were reduced by averaging any data points assigned the same combination of circadian phase and time since last meal (*i.e.*, within subjects and within measures). The impact of condition, circadian phase, and time since last meal on measures of prospective consumption, hunger, satisfaction, and fullness were examined using separate factorial analysis of variance. Each analysis had with one between groups factor: condition (2 levels—severe, moderate); and two within-subjects factors: circadian phase (6 levels—0°, 60°, 120°, 180°, 240°, 300°), and time since last meal (3 levels—1.5 h, 4 h, 6.5 h). All analyses were performed using SUPERANOVA (version 1.11; ABACUS Concepts, Berkeley, CA, USA) with a critical *p* value of 0.05. Data are reported as mean ± SEM.

## 3. Results

### 3.1. Condition

There was no main effect of condition on prospective consumption (F_1,26_ = 3.7, *p* = 0.0671), hunger (F_1,26_ = 0.8, *p* = 0.3873), satisfaction (F_1,26_ = 4.0, *p* = 0.0548) or fullness (F_1,26_ = 3.2, *p* = 0.0864).

### 3.2. Circadian Phase

**Figure 2:**
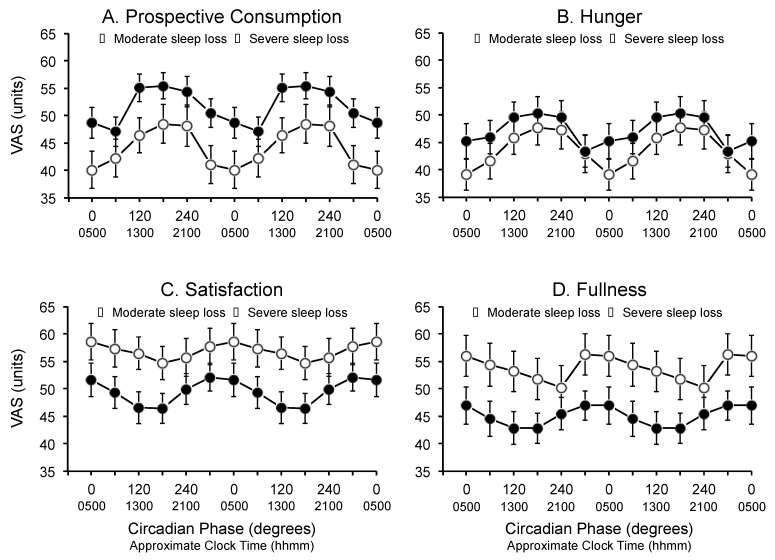
Main effect of circadian phase on (**A**) prospective consumption; (**B**) hunger; (**C**) satisfaction; and (**D**) fullness during the forced desynchrony segment of the protocol for participants in the moderate sleep restriction condition (open circles) and the severe sleep restriction condition (closed circles). Data are double-plotted relative to the circadian nadir (degrees) and are plotted at the midpoint of the bins. Data are mean ± SEM.

There was a significant main effect of circadian phase for prospective consumption (F_5,26_ = 11.4, *p* = 0.0001), hunger (F_5,26_ = 9.0, *p* = 0.0001), satisfaction (F_5,26_ = 2.6, *p* = 0.0275) and fullness (F_5,26_ = 2.6, *p* = 0.0261). Prospective consumption and hunger tended to be highest around the maximum of the core body temperature rhythm (*i.e.*, 180°) and lowest around the minimum of the core body temperature rhythm (*i.e.*, 300°–0°) (Figure 2, top left and right panels). Conversely, ratings for satisfaction and fullness were highest around the minimum of the core body temperature rhythm (*i.e.*, 300°–0°) and lowest around the maximum of the core body temperature rhythm (*i.e.*, 180°–240°) (Figure 2, bottom left and right panels). There were no interactions between circadian phase and condition (*i.e.*, moderate *vs.* severe) for prospective consumption (F_5,130_ = 0.7, *p* = 0.6269), hunger (F_5,130_ = 0.89, *p* = 0.4909), satisfaction (F_5,130_ = 0.56, *p* = 0.7334), or fullness (F_5,130_ = 0.74, *p* = 0.5959).

### 3.3. Time Since Last Meal

**Figure 3:**
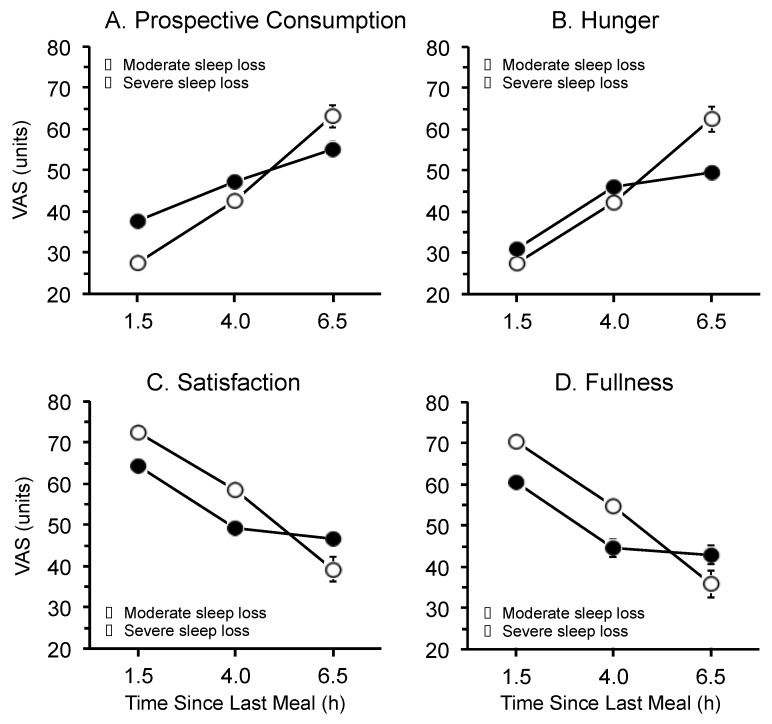
Main effect of time since last meal on (**A**) prospective consumption; (**B**) hunger; (**C**) satisfaction; and (**D**) fullness during the forced desynchrony segment of the protocol for participants in the moderate sleep restriction condition (open circles) and the severe sleep restriction condition (closed circles). Data are mean ± SEM (N.B. in some cases error bars are obscured by the symbols)*.*

Significant main effects of time since last meal were observed for prospective consumption (F_2,52_ = 116.6, *p* = 0.0001), hunger (F_2,52_ = 121.3, *p* = 0.0001), satisfaction (F_2,52_ = 79.4, *p* = 0.0001), and fullness (F_2,52_ = 82.9, *p* = 0.0001) (Figure 3). Prospective consumption and hunger were lowest 1.5 h after consuming a meal, and were highest 6.5 h after consuming a meal. In contrast, satisfaction and fullness were highest 1.5 h after consuming a meal, and were lowest 6.5 h after consuming a meal. There were no interactions between time since last meal and condition (*i.e.*, moderate *vs.* severe) for prospective consumption (F_2,52_ = 1.2, *p* = 0.3034), hunger (F_2,52_ = 0.01, *p* = 0.9902), satisfaction (F_2,52_ = 0.17, *p* = 0.8417), or fullness (F_2,52_ = 0.20, *p* = 0.8213).

## 4. Discussion

In the current study, the influence of circadian phase on hunger and satiety was examined using a 28-h forced desynchrony protocol under two distinct conditions—moderate sleep restriction (e.g., equivalent to 6 h time in bed/24 h) and severe sleep restriction (e.g., equivalent to 4.7 h time in bed/24 h). The results of the study indicate an endogenous circadian rhythm in hunger and satiety, with distinct peaks and troughs across the 24-h day. Specifically, hunger was highest in the biological evening (~17:00 h) and lowest in the biological night (~05:00 h). In contrast, satiety was highest during the biological night (~01:00–05:00 h) and lowest during the biological evening (~17:00–21:00 h). As might be expected, sensations of hunger increased, and sensations of satiety decreased, as time elapsed between meals.

The results from the present study are consistent with expectations based on a previous forced desynchrony study that employed a normal ratio of sleep to wake, *i.e.*, 1:2 [12]. In that study, a strong endogenous circadian rhythm in hunger was observed, with a peak in the biological evening and a trough in the biological morning. In the present study, an opposing rhythm was also observed for satiety, which peaked during the biological morning and was lowest during the biological evening. Together, the results of both studies may help to explain the reduction in appetite that is commonly reported by individuals who work at night [26].

One of the aims of the current study was to determine whether perceptions of hunger and satiety are altered by sleep dose. Two levels of sleep restriction were examined—moderate and severe. A main effect of sleep dose on sensations of hunger and satiety was not observed, but interestingly, mean scores of hunger were higher, and mean scores of satiety were lower, across all circadian phases in the severe sleep restriction compared with the moderate sleep restriction condition. The latter observation suggests that the impact of sleep restriction on sensations of hunger and satiety may be dose-dependent. However, given that only two doses of sleep were examined in the present study and the statistical analyses were inconclusive, further investigation across multiple levels of sleep restriction is required to clarify the relationship between sleep dose and sensations of hunger and satiety.

It is important to consider the potential implications of the results of the present study for individuals who undertake shiftwork. The results clearly show a reduction in the sensation of hunger during the biological night. This finding, coupled with circadian rhythms of appetite hormones (*i.e.*, leptin and ghrelin) [27,28] and gastric acid secretion [29], indicates that the human body is not programed for a nocturnal intake of food [30]. This suggests that the increased risk of metabolic disorders observed in people who perform shiftwork [4,5,6] may be related to factors other than circadian misalignment. One potential explanation is that shiftworkers are required to eat during the night when access to healthy food is limited [31] and/or the time available to prepare healthy meals is restricted [26].

There are some limitations of the current study that should be considered when interpreting the results. First, hunger and satiety were assessed using visual analogue scales rather than assaying blood for hormonal markers (*i.e.*, leptin and ghrelin). The current study was designed to be minimally invasive, so no blood samples could be collected. Second, the study was conducted using a special protocol, with two groups of young, healthy, male participants, in controlled laboratory conditions. As such, caution should be shown in generalising the results beyond these circumstances. Whether or not the results of the current study would be similar if different ratios of sleep to wake were examined or if participants with different characteristics were involved (e.g., females, older, overweight) are empirical questions that are yet to be answered.

## 5. Conclusions

In conclusion, the results of the present study demonstrate an endogenous circadian rhythm in hunger and satiety, with distinct peaks and troughs across the 24-h day. The results of the study provide some evidence to suggest that the impact of sleep dose on sensations of hunger and satiety is dose-dependent, however further investigation is required to confirm this relationship.
